# Does nose spray addiction exist? A qualitative analysis of addiction components in rhinitis medicamentosa

**DOI:** 10.1556/2006.2024.00078

**Published:** 2025-02-11

**Authors:** Lili Lakatos, Borbála Gabriella Koltai, Veronika Ferencz, Zsolt Demetrovics, József Rácz

**Affiliations:** 1 Doctoral School, Health Sciences Division, Semmelweis University, Budapest, Hungary; 2 Doctoral School of Education, ELTE Eötvös Loránd University, Budapest, Hungary; 3 Institute of Education, ELTE Eötvös Loránd University, Budapest, Hungary; 4 Doctoral School of Psychology, ELTE Eötvös Loránd University, Budapest, Hungary; 5 Institute of Psychology, ELTE Eötvös Loránd University, Budapest, Hungary; 6 Institute for Mental Health and Wellbeing, College of Education, Psychology and Social Work, Flinders University, Adelaide, Australia; 7 Centre of Excellence in Responsible Gaming, University of Gibraltar, Gibraltar; 8 Semmelweis University, Faculty of Health Sciences, Department of Addictology, Budapest, Hungary

**Keywords:** rhinitis medicamentosa, nasal decongestant, addiction, qualitative, content analysis

## Abstract

**Background and Aims:**

Nasal congestion is a prevalent symptom often alleviated with over-the-counter nasal sprays containing decongestants. Rhinitis medicamentosa (RM), caused by the overuse of decongestants leading to recurrent mucosal swelling, poses a significant challenge for specialists in managing patients. Despite advancements in understanding, research primarily consists of case series with limited data on its impact on quality of life. This qualitative study aimed to explore the effect of nasal spray overuse on quality of life and identify addiction components among individuals with RM.

**Methods:**

Twenty participants with RM were interviewed by an otorhinolaryngologist and addiction counsellor. The study employed a qualitative approach utilising directed content analysis and revealed eleven categories, classified into addiction components and distinctive features of nasal spray addiction.

**Results:**

The analysis revealed the presence of all Griffiths' addiction components in the identified themes. Additionally, sleep disorders, the feeling of suffocation, side effects, illness identity and psychological effects on nasal congestion significantly impair individuals' quality of life.

**Conclusion:**

This qualitative study identified key components of addiction in nasal spray overuse and suggested that RM might be conceptualised in the DSM-5 category of “Other (or Unknown) Substance-Related Disorders”, considering the lack of psychoactive effects. Nevertheless, in view of the current findings, it also seems to be plausible to examine the phenomenon in the behavioural addiction framework. The study underscores the need for further research and intervention strategies to address the significant impact of RM on individuals' quality of life.

## Background and aims

Nasal congestion stands as a prevalent symptom encountered in otorhinolaryngology practices. A Swedish survey found that as much as 33% of the population is affected by nasal congestion regularly, and 9.5% of people struggle with it daily (Akerlund, Millqvist, Oberg, & Bende, 2006). Patients commonly seek relief using over-the-counter nasal sprays containing decongestants. Nevertheless, the misuse of decongestants can result in recurrent mucosal swelling upon the diminishing effect of the drug, prompting repeated use (Graf, 1997). As a result, non-allergic inflammatory changes develop in the nasal mucosa. The scientific literature describes the condition using various terms, such as rhinitis medicamentosa (RM) (Lake, 1946), chemical rhinitis (Stephens & Boggs, 1968), rebound congestion (Kully, 1945), drug-induced rhinitis (Ramey, Bailen, & Lockey, 2006) and nasal spray addiction (Snow et al., 1980).

According to a survey in the United States, 9% of patients in otorhinolaryngology practices struggled with RM (Feinberg & Feinberg, 1971), and its incidence is continuously increasing (Archontaki et al., 2009). The duration of nasal spray use leading to its harmful effect varies across studies, with reported periods ranging from three days to 4 weeks (Juto & Axelsson, 2014; Morris, Eccles, Martez, Riker, & Witek, 1997; Watanabe et al., 2003); (Akerlund & Bende, 1991).

Despite advancements since its initial description (Feinberg & Friedlaender, 1945), research on this phenomenon primarily comprises case series and histological examinations, with limited data on its impact on quality of life (Scheire et al., 2023). Published theories emphasise morphological and molecular changes, attributing dependence solely to these findings. Recent reviews, such as Zucker et al. (Zucker, Barton, & McCoul, 2019), identify physical dependence as the primary factor contributing to RM, with minimal exploration of psychological aspects. However, Mortuaire et al. state that “this entity could be more appropriately considered to reflect the patient's dependence on topical decongestants to relieve nasal congestion” ((Mortuaire et al., 2013), page 142)). The use of the term “addiction” to describe the withdrawal syndrome characterised by headaches, restlessness, and anxiety following the cessation of nasal drops was initiated by certain authors (Fleece, Mizes, Jolly, & Baldwin, 1984; Snow et al., 1980) and also some case series mention cravings, anxiety and even psychotic episodes associated with withdrawal (De Corso et al., 2020; Fulga et al., 2021; Pearson & Little, 1969; Shure & Harris, 1951; Snow et al., 1980), along with elevated odds of opioid use disorder among RM patients (Patel, Levi, & Brook, 2020). DeCorso et al. (2020) found that smoking habit, a history of psychiatric disorders, drug abuse, insomnia, anxiety disorders and depression were present with a higher prevalence among RM patients (*N* = 68) than the control group (*N* = 51). However, comprehensive research on psychological aspects remains lacking. Qualitative studies on quality of life and patients' experiences with nasal spray decongestant overuse are relatively sparse; so far, only one such study has been conducted (Scheire et al., 2023).

The concept of addiction extends beyond the traditional scope of drug use to include addictive behaviours as well, indicating its roots in a biopsychosocial framework rather than being limited to substances alone. This broader understanding has led to efforts to define addiction in a way that encompasses both substance and non-substance-related behaviours. Griffiths' component model of addiction (Griffiths, 2005) highlights six core elements—salience, mood modification, tolerance, withdrawal, conflict, and relapse—that are essential in identifying the addictive nature of various activities. This model serves as a valuable tool for researchers in assessing the potential for addiction in different behaviours, as demonstrated in the following studies on addictions to exercise (Griffiths, Szabo, & Terry, 2005), work (Andreassen, Griffiths, Hetland, & Pallesen, 2012), internet use (Kuss, Shorter, van Rooij, Griffiths, & Schoenmakers, 2014), sex (Andreassen, Pallesen, Griffiths, Torsheim, & Sinha, 2018) and love addiction (Costa, Barberis, Griffiths, Benedetto, & Ingrassia, 2021).

It has been recognised that nasal decongestants cause physical dependence (Baldwin, 1975; Elwany & Stephanos, 1983; Lacroix, 1989), which, unlike psychoactive substances, is attributed to peripheral rather than central nervous system processes. Nevertheless, in our exploratory study, we aimed to investigate whether the experienced symptoms can be conceptualized in the framework of Griffiths' addiction components model.

## Methods

The study employed a qualitative approach, utilising directed content analysis, and adhered to the COnsolidated criteria for REporting Qualitative research (COREQ) checklist (Tong, Sainsbury, & Craig, 2007).

### Study participants

Patients were recruited face-to-face from a tertiary care Ear-Nose-Throat (ENT) clinic and through social media advertising (20–80%). Written informed consent was obtained from all study participants. Inclusion and exclusion criteria were assessed at the time of application (Table 1). For this study, we included participants who have been using nasal decongestants for at least one year, similar to the inclusion criteria of a previous study (Mehuys et al., 2014), as there is currently no precise information in the literature regarding the duration necessary for addiction to develop.

**Table 1.** The inclusion and exclusion criteria of the study participation

Inclusion criteria	Exclusion criteria
Accepts and signs the informed consent form	untreated allergic rhinitis or rhinosinusitis with severe symptoms
Age above 18 years	malignant tumour of the nasal cavity or parasinuses
Diagnosed with nasal spray dependence by an ENT specialist (based on anamnestic data and physical examination)	concurrent psychoactive substance addiction (e.g. alcohol, opioids, benzodiazepines, psychostimulants
Continuous use of decongestant nasal drops for at least one year during their lifetime AND is an active nasal decongestant user at the time of enrollment	psychotic symptoms, severe mood disorder

A total of 20 interviewees participated out of 28 potentially eligible individuals, comprising six males and 14 females, with ages ranging from 21 to 78 years (mean: 40.6 years, SD = 16.6 years), all identified as white Caucasians. The other 8 candidates did not meet the eligibility criteria. Participants' average continuous decongestant nasal spray use duration was 15.3 years (2–40 years). Most interviews were conducted online (17 out of 20) and lasted 16–52 min. Further detailed demographical, socio-economic and addiction-specific characteristics are provided in Table 2.

**Table 2.** Socio-demographic and descriptive characteristics of the participants

Participant No.	Sex	Age range (years)	Interview duration (min)	Interview type	Highest level of education	Environment	Duration of nasal decongestant use (years)	Agent used	Dose frequency	Smoking status	Considers her/himself as an addict?	Others consider her/him as an addict?	Contact with other nasal spray addicts	Contributing psychological problems	Prior nasal surgery
1	male	40–45	35	O	H	capital	20	O	1 bottle/week	N	yes	yes	no	no	yes
2	female	20–25	22	O	S	capital	10	O	1 bottle/week	N	yes	yes	yes	yes	no
3	male	70–80	26	O	S	suburban	18	O	1 bottle/3 week	?	no	no	yes	no	no
4	female	65–70	52	O	H	urban	30	O	unknown	F	yes	yes	no	yes	yes
5	female	25–30	23	O	H	capital	2	T	1 bottle/week	R	yes	yes	yes	no	no
6	male	25–30	31	O	H	capital	2	T	1 bottle/week	R	yes	yes	yes	no	no
7	female	25–30	23	O	S	capital	13	X	1 bottle/week	C	yes	yes	no	yes	no
8	female	50–55	20	O	S	capital	10	X	3 bottle/month	F	yes	yes	yes	no	no
9	female	50–55	16	O	H	capital	20	X	1 bottle/10 days	N	yes	yes	no	no	no
10	female	40–45	25	O	S	capital	25	O	1 bottle/10 days	F	yes	no	no	no	no
11	female	55–60	20	O	S	urban	40	X	1 bottle/5–6 days	?	yes	yes	no	no	yes
12	male	40–45	27	O	H	capital	25	O	1 bottle/month	?	yes	yes	no	yes	yes
13	female	25–30	31	IP	S	capital	13	T	1 bottle/4–5 days	?	yes	yes	no	yes	no
14	male	20–25	19	O	H	urban	7	O	1 bottle/5–6 days	N	yes	yes	yes	no	no
15	female	40–45	45	O	H	capital	3	X	1 bottle/2 weeks	N	no	no	no	no	no
16	male	25–30	25	O	H	capital	3	T	1 bottle/2–3 weeks	N	yes	yes	yes	no	no
17	female	25–30	33	O	S	capital	10	X	unknown	N	yes	yes	yes	yes	no
18	male	55–60	31	IP	H	capital	40	T	1 bottle/5–6 days	F	yes	no	no	no	yes
19	female	25–30	31	IP	S	suburban	10	O	1 bottle/2–3 days	N	yes	yes	yes	yes	no
20	female	40–45	33	O	S	urban	5	X	1 bottle/week	F	yes	yes	no	yes	no

*Notes*: The interview type was online (O) or in-person (IP). The highest level of education was distinguished as secondary (S) or higher (H). The decongestant agent administered was identified as oxymetazoline (O), xylometazoline (X), or tramazoline (T), and smoking status was classified as never (N), rarely (R), former (F), or current (C).

### Data collection

The first author, a female otorhinolaryngologist and addiction counsellor (LL), with qualitative research training, managed participant recruitment, constructed and pilot-tested the interview guide (see Appendix) and conducted the interviews. Another physician diagnosed RM beforehand to ensure impartiality. The interviews were conducted in Hungarian, as all participants were native speakers. Depending on the participant's preference, sessions took place via video call or face-to-face in a private consulting room. Each session involved only the interviewer and the participant. Participants were assured that the interviewer was solely engaged in a research capacity and that their interactions would not impact their care. Each participant was interviewed according to the same guide. No repeat interviews were carried out. To acknowledge and appreciate the time and effort contributed by participants, each patient received a voucher valued at 5000 HUF (approximately 13 Euros).

During the semi-structured interviews, both open-ended and directed questions were posed to inquire about how participants experience their addiction, the emotions it evokes in them, the difficulties they face, how it affects them and their environment, their experiences with quitting, and what kind of help they have sought so far. We compiled the questions based on Griffiths' addiction component model to cover all aspects.

Interviews, ranging from 16 to 52 min (median: 26 min), were audio-recorded and transcribed verbatim using Alrite© speech recognition software (Régens PLC, Budapest, Hungary). No field notes have been made. Transcriptions were meticulously cross-checked against the original recordings to ensure accuracy.

### Researchers' background and reflexivity

LL had no prior acquaintance with the participants. She brought both professional knowledge of RM from her medical background and personal experience with nasal spray addiction throughout her lifetime. This information was available to the participants before study participation. The second and third authors (BGK, VF) are doctoral students in education and psychology, respectively. JR is a practising psychiatrist, and ZD is a clinical psychologist; they are both addiction treatment experts. They do not have previous experience with RM patients nor have a personal addiction history. As a practising ENT specialist, LL regularly attends to patients afflicted with RM and faces the challenges arising from the absence of efficacious therapeutic interventions. LL suspects that addictive elements may be present in this phenomenon and her own experience with nasal spray dependency further supports this hypothesis. By engaging in self-reflexivity and authors working together as a team, she aimed to minimise the impact of her biases and assumptions on the interpretation of data.

### Data analysis

Directed content analysis (Downe-Wamboldt, 1992; Hsieh & Shannon, 2005) is a qualitative research method that begins with a theory or prior research findings to guide the initial coding process. The primary purpose is to validate or extend an existing theoretical framework. Using existing theory, researchers begin by identifying key concepts or variables as initial coding categories (Potter & Levine-Donnerstein, 1999). The theoretical framework of this study is based on Griffiths' addiction components model. The components served as initial coding categories. We applied these codes to the data, highlighting relevant text and categorizing it accordingly. Any data not fitting into the existing categories is given a new code. The analysis continued by examining the data to see if subcategories were needed or if new categories emerged.

The first author primarily analysed all transcripts. The authors, functioning as an interdisciplinary research team, collectively participated in data analysis. Regular meetings were held every two weeks, during which the identified codes and themes were reviewed and refined.

### Ethics

This study was carried out following the Declaration of Helsinki and approved by the Hungarian Medical Research Council- Scientific and Research Ethics Committee (BM/3332-1/2023).

## Results

**Fig. 1.**
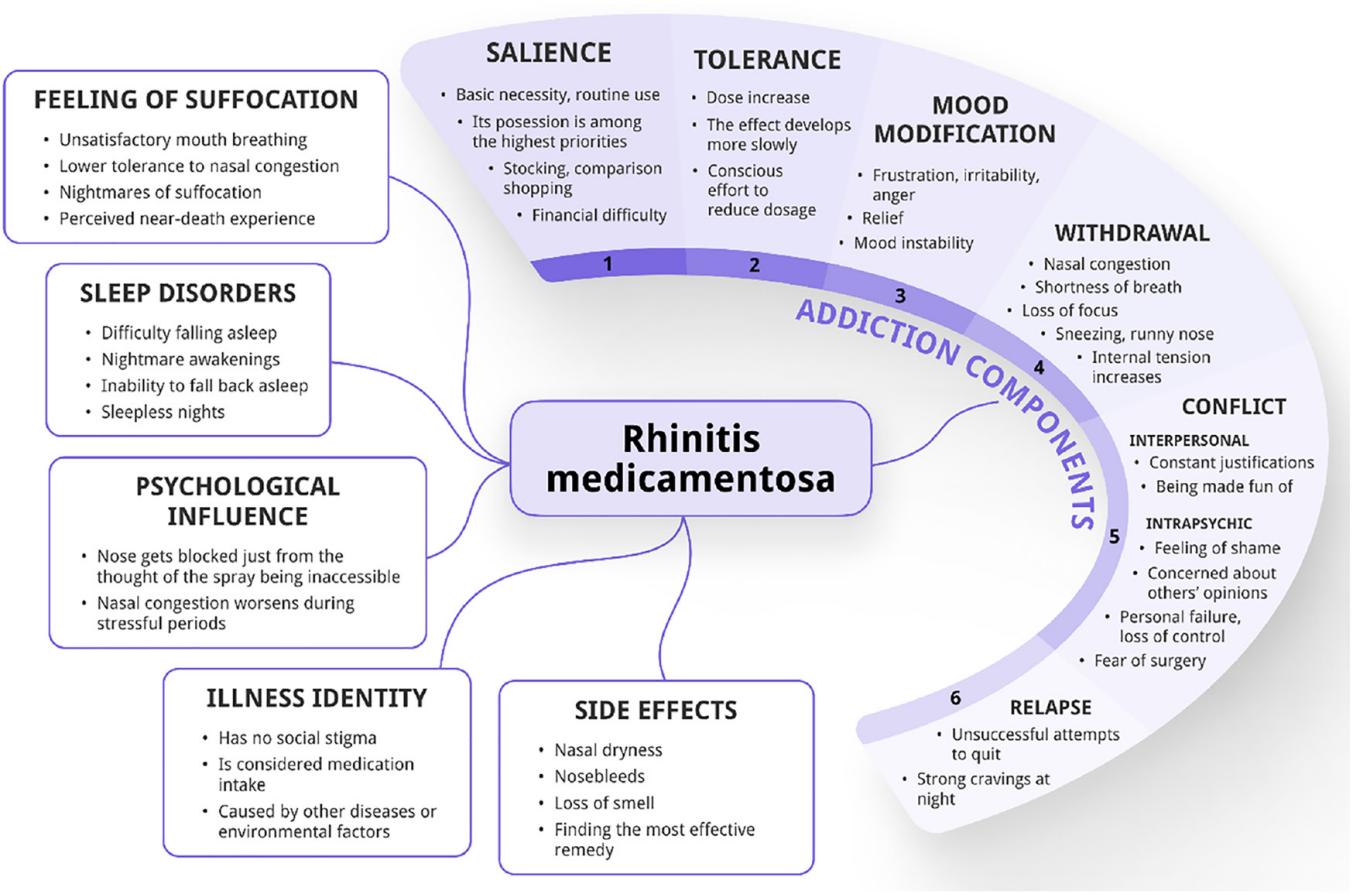
The figure illustrates the different categories and their relationships that arose during the analysis. The numbered sequence of the addiction components also indicates their emphasis in the interviews

Eleven categories were identified from the analysis of the interview transcripts, which we categorised into two main groups: addiction components and distinctive features of nasal spray addiction (refer to Fig. 1). All six categories of Griffiths' addiction components model were identified in the addiction components group. The frequency of each category's occurrence in the interviews is summarized in Table 3 to illustrate the emphasis of each category. The illustrative quotes used for each category are provided in Table 4.

**Table 3.** The table contains categories representing addiction components and the distinctive features of nasal spray dependency, which were identified from the interview content

Patient Number		Prevalence of categories among participants																				
		01	02	03	04	05	06		07	08	09	10	11	12	13	14	15	16	17	18	19	20
**Addiction components**																						
Salience		X	X	X	X	X		X	X	X	X	X	X	X	X	X	X	X	X	X	X	X
Mood modification		X	X		X	X		X	X	X		X	X	X	X	X	X	X	X	X	X	
Tolerance		X	X	X	X	X		X	X	X	X	X	X	X	X	X	X	X	X	X	X	X
Withdrawal		X	X	X	X	X		X	X		X	X	X	X	X	X	X	X	X	X	X	X
Conflict:	intrapsychic.	X	X		X	X		X		X		X		X	X	X	X	X	X	X	X	X
	interpersonal	X	X		X	X		X	X						X	X		X	X		X	X
Relapse		X	X		X			X				X	X	X	X	X	X		X	X	X	X
**Distinctive features**																						
Feeling of suffocation		X	X	X	X	X		X		X		X	X	X	X	X	X	X	X	X	X	X
Sleep disorders		X	X	X	X	X		X	X	X	X	X	X	X	X	X	X	X	X	X	X	X
Specific side effects		X	X	X	X	X		X	X	X	X			X		X	X	X	X	X	X	X
Psychological influence		X	X		X					X				X		X	X				X	
Illness identity			X	X	X				X	X	X		X	X	X		X		X		X	X

**Table 4.** Illustrative quotes representing the categories and subcategories identified in the directed content analysis of the interview transcripts

Category	Subcategory	Participant code	Quote code	Quote
Salience	Necessity	P07	Q01	*“So, for me, the nasal spray was like the wallet or the phone.”*
	Comparison shopping	P18	Q02	*“I really don't want to let go of this much money, to be honest. I'm going to check online now, maybe even order it, because it is already around 3,000 [in HUF, ∼7,6 Euro]. But I'm getting it for 2,500 [in HUF, ∼6,3 Euro] at wholesale price.”*
	Highest priority	P04	Q03	*“Well, especially while driving when it got blocked… I often pulled over, leaned back on the seat, and used the drops. And when it was fine, I continued driving, and then everything was fine.”*
Tolerance	Dose increase	P01	Q04	*“During my last illness, I felt that all the symptoms had passed except for this nasal congestion, and I was using it a lot at that time. I mean, really, I sprayed three times into each nostril each time, and I did this, I think, about eight times a day.”*
	Efforts to reduce dosage	P15	Q05	*“So, I tried to reduce it; I didn't use the spray constantly, but only when the situation became unbearable, and I really couldn't breathe.”*
		P06	Q06	*“Of course, I'm trying to use it less, and now I don't spray it eight times a day like before, just way less often. So, I don't feel like it's a problem.”*
Mood modification	Anger, frustration	P01	Q07	*“I was actually angry, I was mad about this, that it's happening again. Again I have to spray, again there's no nasal spray nearby, again I'm tired, again I woke up in the morning feeling like a squeezed lemon.”*
	Relief	P16	Q08	*“I'm very happy when it clears up. That really cheers me up.”*
	Mood instability	P11	Q09	*“When my nose is really blocked, I feel a bit tense because of it. I mean, it's the feeling itself that makes me tense. And if I, use the drops, that tension just goes away. So…but I think it's because I'm finally able to breathe.”*
Withdrawal	Inner tension increases	P13	Q10	*“I don't just use it when it's blocked, but when I know it's going to be because I've experienced what it's like when it's going to block in half an hour. And then I must use it beforehand anyway, otherwise I get this terrible feeling that I'm very nervous, that I can't breathe”.*
	Loss of focus	P06	Q11	*“Last time, we were at the cinema, and I didn't take it with me. I couldn't even concentrate on the movie because I couldn't breathe, and it simply took away my attention.” (Participant 06)*
Conflict – interpersonal	Being made fun of	P01	Q12	*“In our friends' group, well, let's just say it's kind of a joke, but not in a mean way at all, and it's nothing I can't handle.”*
	Justifications	P19	Q13	*“Once, they already made a comment to me at the pharmacy when I had just been there for nasal spray, asking why I was buying so much. And then I would always lie, saying it wasn't for me, because I didn't dare to admit the truth.”*
Conflict – intrapsychic	Loss of control	P20	Q14	*“Honestly, I have a very strong will. If I set my mind to something, I get it done, but I can't handle this. (…) So, in the middle of the night, when I felt like I was suffocating, I lost control and used the spray.”*
	Feeling of shame	P13	Q15	*“I feel ashamed that I still haven't been able to quit this, and that I pay for this increasingly expensive nasal spray every two weeks.”*
	Concerned about others' opinions	P14	Q16	*“I didn't want to expose myself to people making remarks like, 'you're nasal spray addict'.”*
Relapse	Unsuccessful attempts to quit	P18	Q17	*“It was a little over half a year when it seemed like I didn't need it, but somehow the issue returned.”*
	“Back to square one”	P10	Q18	*“Every time I feel like I could quit, I get sick again, and I can't breathe. I end up using the nasal spray again, and it's a vicious cycle.”*
Feeling of suffocation	Unsatisfactory mouth breathing	P04	Q19	*“There were those who said, 'Just open your mouth and take a breath.' Well, it's not that simple.”*
		P19	Q20	*“Once I tried it [referring to the attempt to quit], I managed for half a day because probably things inside had already died off so much, or I don't know, it was like I couldn't even get air through my mouth, it was so blocked.”*
	Nightmares of suffocation	P10	Q21	*“I had these nightmares, that they would kidnap me and duct-tape my mouth. And then I would die of suffocation.”*
	Lower tolerance to nasal congestion	P12	Q22	*“I think I'm dependent. The question is whether I'm dependent on the substance, or if I'm dependent on breathing itself, then I'd rather say I'm dependent on nose breathing.”*
Sleep disorders	Sleepless nights	P16	Q23	*“My nose gets so blocked at night that it really frustrates me, and I get anxious. I can't fall back asleep, even when it clears up.”*
	Difficulty falling asleep	P15	Q24	*“I especially don't tolerate nights well because, during the day, one can still manage, but at night, not being able to sleep, that's just terrible. And I can't breathe through my mouth, so I can't sleep while breathing through my mouth.”*
Psychological influence	Just to think of it causes blockage	P08	Q25	*“So, I noticed that if I don't have the nasal spray, then my nose immediately gets blocked. Really, one can become fixated on this thing, like any addict.”*
Side effects	Loss of smell	P20	Q26	*“I really want to finally breathe in, to take in fresh air completely through my nose and into my lungs, because even if I inhale, the breathing is not the same as it used to be. I don't feel the smells the same way; I feel very restricted.”*
Illness identity	No stigma	P10	*Q27*	*“I honestly don't think anyone cares. This isn't something noticeable, something that smells bad, something… well, it damages my body, okay, but it's still not a drug. So, nasal spray is not something that is socially forbidden.”*
	It is a medication	P13	*Q28*	*“Everyone has a different attitude towards this because it's a medication, and otherwise, I can't breathe.”*
	Environmental factors	P12	*Q29*	*“If the humidity is high, then the mucous membrane is more sensitive.”*

### Addiction components

#### Salience

When a particular activity becomes salient, it gains the foremost focus in an individual's life, influencing their thoughts, emotions, and actions. This is particularly evident when the individual faces situations where they cannot access the addictive substance or behaviour, causing the addictive activity to dominate their attention. Carrying nasal spray becomes a necessity in their bags, and they stockpile supplies everywhere to avoid running out accidentally.

For them, the nasal spray is not only a physical object but a crucial component of maintaining their quality of life and a significant part of their identity (see quote Q01 in Table 4). Its possession is among the highest priorities in their life.

Financial difficulties often arise from funding their addiction, leading them to shop around pharmacies seeking the cheapest option (Q02). This suggests that individuals face a larger existential issue, weighing the price of maintaining comfort against how much they are willing to sacrifice for their livelihood to avoid discomfort.

Manipulation with packaging is typical (e.g. Participants 7, 12), such as transferring nasal drops into spray bottles to enhance dispersion (e.g. Participant 2). This practice is typical for stretching resources, as drops are cheaper. Some may interrupt their work to take a dose or request favours from others to go to the pharmacy while they cannot (e.g. Participants 8 and 17). Participant 04 often paused her travel (Q03), which suggests that it is more important to regain control over breathing than any other activity.

#### Tolerance

Most of our interviews revealed that nasal spray users may initially increase doses and experience a slower onset of action. However, over decades, a stable usage pattern is established, with dose escalation mainly occurring during upper airway infections (Q04). A conscious effort to reduce the dose for harm reduction can be observed once individuals recognize their issue, pushing the limits of their tolerance (Q05). Participant 06 believes that dose reduction is the key to addressing the problem and tends to relativise its seriousness (Q06).

This reflects an attempt to manage the problem, where participants actively seek solutions to their condition. The fact that they consciously try to reduce their usage indicates an understanding of the risks and an internal struggle to regain control over their lives. This effort can also be interpreted as taking responsibility and a step toward recovery.

#### Mood modification

The use of nasal spray not only alleviates physical symptoms but also has a profound impact on individuals at an emotional and psychological level. Both ends of the emotional spectrum emerged from the data: positive feelings associated with using nasal spray and negative emotions triggered by the cessation of its effects, such as frustration, irritability, anger, aggression, and inner tension (Q07). This is primarily due to the individual repeatedly confronting their dependency and the resulting sense of helplessness.

Positive emotions related to nasal spray use included relief, the joy of improved nasal breathing, a sense of freedom, gratitude, and a feeling of security (e.g., Q08). From an existential perspective, this joy creates the illusion of experiencing temporary freedom; the individual may feel liberated for a short period, but this sense of freedom is fleeting. This duality highlights the fluctuating emotional experiences induced by dependency, where relief and frustration continuously alternate. This instability also affects one's sense of identity, as individuals may feel they are not in control of their emotions, which seem to shift depending on their use of nasal spray (Q09).

#### Withdrawal

In our interviews, most withdrawal symptoms are associated with nasal obstruction. The onset of nasal congestion is often so distressing and tension-inducing that they sometimes use nasal spray even when there is no congestion to prevent it from occurring later (Q10).

Participants are aware that the loss of focus, drowsiness, dullness, and sleep deprivation caused by a blocked nose significantly negatively impact their well-being, making it difficult to carry out their daily tasks (Q11). This experience points to a sense of existential loss of freedom: individuals cannot fully engage in life's experiences because their physical limitations prevent them from freely enjoying them.

In addition to nasal congestion, sneezing, runny nose, dry mouth, sore throat, headache, and nausea are commonly experienced (e.g. Participants 1, 13, 16).

#### Conflict

Interpersonal and intrapsychic conflicts were reported, so we divided these into subcategories. As shown in Table 3, interpersonal conflicts occurred less frequently, while the latter was more common among the participants. Society is often unfamiliar with this phenomenon and may react with scepticism or even joke about it when encountering someone with this condition (Q12). However, some of them sense disapproving looks, as Participant 19 reported (Q13).

Since RM does not directly harm those around them, there is no pressure from others for the person to quit. While they may desire to quit, they feel unable to, viewing it as a personal failure. They experience a sense of loss of control over this aspect of their life (Q14), dominated by self-blame and guilt (Q15).

Fearing judgment or feeling ashamed, many treat their addiction as a taboo and keep it hidden (Q16). Sometimes, they must explain themselves, clarifying that their sneezing or nasality is not due to illness or crying. They may also feel embarrassed in their relationships, such as from snoring or sleeping with their mouth open (e.g. Participants 7, 10, 13), which may reflect fragility in self-esteem.

#### Relapse

Most of our interviewees reported a history of multiple attempts to quit (Q17). Relapses often coincide with illnesses, such as the common cold (Q18). Relapse not only results in the reinstatement of physical dependence but also causes significant emotional and psychological distress, potentially leading to self-criticism and hopelessness. The ongoing struggle to overcome addiction can exacerbate issues of self-esteem and self-acceptance.

### Distinctive features of nasal spray addiction

We present separately those categories that also manifested prominently during the interviews but did not fit neatly within the framework of addiction components.

#### Feeling of suffocation

Despite being able to breathe through the mouth undisturbed, participants reported experiencing a sensation of suffocation due to nasal congestion. Mouth breathing is unsatisfactory for them; they feel like they are running out of air (Q19, Q20). Nightmares related to suffocation torment them, and some compare waking up with a blocked nose to a near-death experience (Q21). This suggests that restriction of breathing evokes anxiety related to the loss of freedom, or even death, and demonstrates how nasal congestion infiltrates the subconscious mind.

At such times, their sole and most important task is relieving nasal congestion, even if it means squeezing out the last drop from the bottle. Several participants expressed that they do not need the medication itself but rather the ability to breathe through their nose. They would gladly forget about the nose spray if an alternative could achieve the same effect (Q22). This raises a critical question about the nature of dependency, whether it is the substance itself or the ability to breathe freely that drives their addiction.

#### Sleep disorders

Nearly all interviewees reported relying on the nasal spray for restful sleep. Using nasal spray becomes a nightly ritual that ensures a sense of control and security before sleep. Without it, they have trouble falling asleep, or they wake up during the night, making it challenging to return to sleep (Q23).

Quit attempts are often unsuccessful due to nighttime nasal congestion, as it is less tolerable than during the day. At night, the absence of daytime distractions makes the discomfort of nasal congestion feel more intense and intolerable. The frustration of Participant 15 at being unable to tolerate nighttime congestion (Q24) highlights how essential sleep is to their mental and emotional well-being.

#### Psychological influence

Some participants noticed that even the thought of not having their nasal spray being inaccessible caused nasal congestion (Q25). The quote reflects that the body reacts to the expectation of distress, highlighting the role of mental anticipation in the onset of physical symptoms.

When congestion begins, “feelings of anxiety similar to claustrophobia” (e.g. Participant 04) or “the beginning of a panic attack” (e.g. Participant 20) may arise. Both are marked by feelings of loss of control and feelings of entrapment, indicating that nasal congestion is not only a physical blockage but also an emotional trigger for deep-rooted fears. They also react worse to nasal congestion during stressful periods.

#### Side effects

Participants reported local damaging effects such as nasal dryness, burning sensations, discharge, and nosebleeds, which caused significant discomfort. As a result, they experimented with various medications to find the most effective option with the fewest side effects. Once they had it, they cling to it – this topic came up in almost every interview. Many feel the worsening of their sense of smell as a harmful consequence (Q26), and because of this, they also do not perceive the taste of food and do not enjoy eating (e.g. Participant 5).

#### Illness identity

Most of our interviewees admitted that society is less judgmental about RM compared to alcohol or drug addiction, as it does not carry the same stigma and is often not taken seriously (Q27). People are more understanding, lenient, and helpful towards nasal spray users.

For some, nasal spray use has become a normalized, almost medicalized, part of their daily routine, resembling a necessary medication intake (Q28). Viewing the spray in this way, rather than as an addiction, helps justify its continuous use. This reframing may act as a coping mechanism, allowing participants to view their dependency through the lens of health management rather than addiction. This illustrates the complexity of illness identity, where participants may fluctuate between seeing themselves as patients managing a medical condition and individuals struggling with dependency.

The nasal congestion caused by the spray itself is often attributed to external factors or underlying physical conditions such as allergies, a deviated septum, and external humidity (Q29).

## Discussion

### Interpretation of findings

Our analysis revealed individuals' frustrating and troublesome experiences with nasal spray overuse. From our results, all themes covering the addiction criteria of Griffiths' addiction components model have been identified from the interviews with the participants. The topics of salience, mood modification, withdrawal, tolerance, conflict, and relapse – as core dimensions of addiction - were represented in our sample, so it is justifiable to say that an addiction-like condition develops in patients with RM. We must emphasise that this assumption is based only on observations and lacks further evidence. Our interviewees have been using decongestant nasal sprays for at least two years, so this suggests that over this period, not only the physical but also the psychological aspects of addiction emerge. Furthermore, sleep disorders, the feeling of suffocation, side effects, illness identity and the psychological influence on nasal breathing are significant concerns for patients and should be considered during treatment. The patients regard this as an illness, which furthermore contributes to the persistence of the addiction.

The frequency of each category's occurrence across the interviews is summarised in Table 3. According to our findings, not all six components appeared to have equal emphasis in the results. Salience and tolerance were identifiable in all participants, whereas interpersonal conflict and relapse were mentioned less frequently. Is this significant, and what does it imply if a component is missing? In previous studies, for instance, in questionnaires measuring social media addiction (Fournier et al., 2023), the highest mean scores were also reported for the components of salience and tolerance. In contrast, in the case of mukbang addiction (Kircaburun et al., 2021), the highest score was recorded for the item assessing mood modification. In a recent study on smartphone addiction (Jameel, Shahnawaz, & Griffiths, 2019), the component of relapse was not explicitly reported by any participants. The explanation may be that as the nature and impact of specific activities change, different aspects of addiction become more prominent. The dominance of particular components did not affect the criterion validity or reliability of the questionnaires. However, Griffiths (Griffiths, 2005) does not take a definitive stance on the dominance of the six components in an addicted individual or whether a phenomenon can still be considered an addiction in the absence of any component. In our sample, several participants had not yet attempted to quit, which may explain the underrepresentation of the relapse component. Among our interviewees, some were each other's life partners. This situation may have influenced their perceptions regarding social relationships and conflict situations.

During the interviews, several participants expressed that they do not need the nose spray itself but rather the ability to breathe through their nose. The excessive attachment is not truly linked to the substance itself but to the effect it produces. The effect induced by the mentioned substance is the alleviation of withdrawal symptoms, which is why we cannot consider it a classical substance use disorder. Furthermore, research findings have not yet confirmed the association of nasal decongestant drugs with the activation of specific reward structures in the brain. In our opinion, two factors contribute most to the participants' strong attachment to nasal breathing. Firstly, these pharmaceuticals cause significant vasoconstriction in the nasal mucosa, enable nasal breathing to reach such a good level that it surpasses physiological conditions (Pertuze, Watson, & Pride, 1991) and induce subjective pleasant sensations by free nasal breathing, easily filled lungs, and airflow sensation in the nasal passages. On the other hand, the fact that not every nasal spray user becomes dependent raises the suspicion that there may be individual predispositions to develop an addiction. Most of our interviewees reported lower tolerance to nasal congestion, or some had experienced a particular event where they associated it with a specific stressful situation (for example, experiencing significant discomfort during an upper respiratory illness). Many participants reported that nasal congestion might trigger anxiety, stress, or even the “beginning of panic attacks” for them. Some people consider their excessive nasal spray use as taking medication, which could be a defence mechanism – rationalization – to cope with the anxiety of realizing their addiction.

A recent qualitative study on RM by Scheire et al. (2023) focused on the lived experiences of long-term nasal decongestant users and presented a grounded theory in which the core theme was the indispensability of nasal spray. Their results highlight how nasal spray permeated their everyday lives and the desperate need for the substance to maintain satisfying nasal breathing. Although there are many similarities between our findings, they had no focus on the addiction itself. Additionally, they also mention that it remains “unclear whether RM must be considered a true addiction.” (page 608., Sheire et al., 2023.)

According to the fifth edition of the Diagnostic and Statistical Manual of Mental Disorders (DSM-5) (American Psychiatric Association, 2022), Substance-Related and Addictive Disorders encompass two categories: Substance-Related and Non-Substance-Related Disorders. A subcategory of the former is “Other (or Unknown) Substance-Related Disorders,” which pertains to problematic patterns of use of a non-intoxicating substance not classifiable under traditional categories such as alcohol, caffeine, cannabis, hallucinogens, inhalants, opioids, sedatives, hypnotics, anxiolytics, stimulants, or tobacco, and leading to clinically significant impairment or distress. This category includes anabolic steroids, antihistamines, and non-steroidal anti-inflammatory drugs, among others. Therefore, nasal decongestants lacking psychoactive effects (when used at average doses) may be considered under this category.

However, if we consider it a Non-Substance-Related Disorder, we must examine whether it aligns with the criteria for behavioural addiction. Our results showed that Griffiths' definition of addiction components is fulfilled. Alternatively, according to the proposed definition by Brand et al. (Brand et al., 2020) for the new ICD-11 category of “other specified disorders due to addictive behaviours”, three meta-level criteria are outlined: (1) empirical evidence demonstrates that the potential addictive behaviour is clinically relevant, leading to negative consequences and functional impairments in daily life; (2) current theories on addictive behaviours appropriately describe and explain the phenomenon of the candidate addictive behaviour; (3) empirical evidence suggests that psychological (and neurobiological) mechanisms involved in other addictive behaviours are also valid for the candidate phenomenon. Our results demonstrated significant functional impairment, with marked negative consequences primarily affecting the individuals themselves. In our study, we examined Griffiths' model and found it applicable. However, further investigations are warranted in the future, particularly concerning the third criterion, by applying sufficient and rigorous methods and assessment instruments.

The question may arise as to why we did not apply the DSM-5 addiction criteria instead of the components model in our study. Although the DSM-5 Substance Use Disorder criteria could have been considered, we ultimately decided against it because decongestant nasal sprays do not fall into any of the 10 drug classes listed in the DSM-5, as they lack psychoactive effects. The condition is more relatable to behavioural addictions.

### Strength and limitations

The primary advantage lies in investigating a distinctive and under-examined sample, offering detailed, idiographic perspectives into their experiences and understanding. Our study is inherently qualitative, thus its results are exploratory and stem from a small, non-representative sample, so they may not necessarily be generalisable to all individuals with RM. We interviewed individuals who had been using nasal sprays for over a year. Therefore, the experiences of those who have been using them for a shorter period are not included in this research, but this could have influenced the results. Despite efforts to maintain reflexivity during analysis, we acknowledge potential bias from pre-understanding. Despite being personally affected by the condition, LL refrained from sharing personal experiences, opinions, or emotions to prevent influencing participant responses and maintain objectivity. We should also mention the limitations of Griffiths' addiction components model. In a recent study by Fournier et al. (2023), network analysis results revealed that the six components of addiction did not form a cohesive, unitary construct. Instead, they clustered into a bidimensional construct, where the first dimension (salience and tolerance) showed no association with any measures of psychopathological symptoms included in their study. This finding suggests that the conceptualization and assessment of behavioral addictions may need to be reconsidered, as the current criteria could lead to the over-diagnosis of non-pathological behaviors. There is currently an active debate surrounding the model. Amendola (2023) re-analyzed Fournier et al.’s data and concluded that their observations were unjustified. However, Fournier et al. (2024) continue to defend their findings, keeping the debate unresolved. An additional limitation is that not all questions in the interview guide were open-ended. However, participants still provided detailed responses, as reflected in the length of the interviews. When answers were brief, we prompted further elaboration with follow-up questions.

## Conclusion

RM is frequently referred to as an addiction, although its definitive classification as such remains uncertain. This qualitative study identified the critical components of addiction according to Griffiths' criteria. It made an attempt to categorise this phenomenon as a unique, substance-related form of behavioural addiction. Besides that, it was also prominent that sleep disorders, feelings of suffocation, and side effects also have significant impairments in the individual's quality of life. Our research could be valuable for clinicians and pharmacists in gaining a deeper understanding of their patients and approaching them with greater empathy. Future research may focus on intervention development, such as incorporating cognitive-behavioural therapy and recovery-oriented addiction practices. Involving experiential helpers or support groups in nasal spray addiction recovery could expand therapeutic options and complement existing strategies.

## Appendix Interview Guide

### Interview Structure:

Part 1: Providing information about the purpose and process of the study and obtaining informed consent.
Part 2: Obtain socio-demographical information and conduct the interview using the following guiding questions.
Part 3: Concluding the interview, summarising, requesting feedback, discussing, and sharing future results.

### Questions:

- Since when have you been using the nasal spray, and how did the addiction start for you?
- What are your feelings regarding your addiction?
- How do you perceive yourself? Do you consider yourself addicted?
- Do others perceive you as addicted?
- Does nasal spray use cause difficulties in your life?
- To what extent does nasal spray use define your daily activities and thoughts? For example, concerns about having enough for the weekend and frequent visits to the pharmacy.
- How does your nasal spray addiction affect the quality of your sleep?
- Does your addiction affect your social relationships? For example, have arguments, shame, guilt, or secrecy occurred because of it?
- Does your addiction cause financial difficulties?
- Have you felt that you lost control over nasal spray use? (Considering dosage and frequency)
- Does nasal spray use bring any benefits to your life?
- How does your mood change about nasal spray use?
- Have you attempted to quit? If so, what method did you use?
- Have you experienced withdrawal symptoms?
- When you recognised the problem, did you seek help? Who did you turn to?
- Have you experienced psychological problems or other addictions in your life?
- Is there anything else that I haven't asked about but came to your mind?
